# The insulin receptor substrate Chico regulates antibacterial immune function in *Drosophila*

**DOI:** 10.1186/s12979-016-0072-1

**Published:** 2016-05-01

**Authors:** Sarah McCormack, Shruti Yadav, Upasana Shokal, Eric Kenney, Dustin Cooper, Ioannis Eleftherianos

**Affiliations:** Insect Infection and Immunity Laboratory, Department of Biological Sciences, Institute for Biomedical Sciences, The George Washington University, 800 Science and Engineering Hall, 22nd Street NW, Washington, D.C., 20052 USA

**Keywords:** *Drosophila melanogaster*, Long-lived mutant, *Chico*, Ageing, Infection, Insect pathogen, *Photorhabdus*, Innate immunity

## Abstract

**Background:**

Molecular and genetic studies in model organisms have recently revealed a dynamic interplay between immunity and ageing mechanisms. In the fruit fly *Drosophila melanogaster*, inhibition of the insulin/insulin-like growth factor signaling pathway prolongs lifespan, and mutations in the insulin receptor substrate Chico extend the survival of mutant flies against certain bacterial pathogens. Here we investigated the immune phenotypes, immune signaling activation and immune function of *chico* mutant adult flies against the virulent insect pathogen *Photorhabdus luminescens* as well as to non-pathogenic *Escherichia coli* bacteria.

**Results:**

We found that *D. melanogaster chico* loss-of-function mutant flies were equally able to survive infection by *P. luminescens* or *E. coli* compared to their background controls, but they contained fewer numbers of bacterial cells at most time-points after the infection. Analysis of immune signaling pathway activation in flies infected with the pathogenic or the non-pathogenic bacteria showed reduced transcript levels of antimicrobial peptide genes in the *chico* mutants than in controls. Evaluation of immune function in infected flies revealed increased phenoloxidase activity and melanization response to *P. luminescens* and *E. coli* together with reduced phagocytosis of bacteria in the *chico* mutants. Changes in the antibacterial immune function in the *chico* mutants was not due to altered metabolic activity.

**Conclusions:**

Our results indicate a novel role for *chico* in the regulation of the antibacterial immune function in *D. melanogaster*. Similar studies will further contribute to a better understanding of the interconnection between ageing and immunity and lead to the identification and characterization of the molecular host components that modulate both important biological processes.

## Background

Ageing involves a large number of complex changes in the physiology of animals. Most of these changes lead to general decline in the fitness of the animal, deterioration of many vital functions, and a subsequent exponential increase in mortality [1]. The constant threat posed by infectious microbes has made the host immune response an essential feature across phyla [2, 3]. The immune system plays a pivotal role in ageing, age-associated disorders and longevity determination. Earlier reports have also indicated that ageing is correlated with a decline in immune functions [4]. Immune deficiencies are associated with pathologies, many of which increase in frequency with age. Ageing individuals suffer increased mortality upon infection due to reduced capacity to activate immune mechanisms in response to microbial challenge [5].

Deterioration in immune function with age has been observed in both invertebrate and vertebrate organisms. Invertebrate model organisms are excellent systems for the study of complex biological processes. The common fruit fly, *Drosophila melanogaster*, has emerged as the organism of choice to investigate the regulation of immunity and ageing signaling pathways that share extensive similarity to those of mammals [6–8]. In addition, *D. melanogaster* is devoid of an adaptive immune system, and thus it is an ideal model to elucidate pristine innate immune defenses [9]. The genetic tools and genomic information available in *D. melanogaster* allow the molecular and physiological dissection of the interaction between ageing and immunity [10].

The Insulin/Insulin-like Growth Factor Signaling pathway (IIS) is an evolutionary conserved pathway that regulates ageing [11]. Mutations in certain genes that decrease IIS signaling can significantly extend life span in diverse species including *D. melanogaster*. The effect of IIS on life span has been attributed to increased resistance to oxidative stress and increased activity of cellular detoxification pathways [12, 13]. Chico is the *D. melanogaster* homolog of vertebrate insulin receptor substrates that modulates IIS. Mutations in *chico* substantially affect cell growth and proliferation, but they have little effect on cell fate and differentiation and no effect on cell viability [14, 15]. Previous studies have shown increased survival of long-lived *D. melanogaster chico* mutant flies in response to bacterial infection [16]; however, enhanced survival ability was not due to significant upregulation of antimicrobial peptide (AMP) genes in the mutant flies.

Here we have expanded these studies by testing the immune response of *chico* loss-of-function mutant flies against pathogenic *Photorhabdus luminescens* and non-pathogenic *Escherichia coli* bacteria. *P. luminescens* are remarkable bacteria because they possess two contrasting lifestyles, mutualistic and pathogenic [17]. They live in mutualism with their nematode vector *Heterorhabditis bacteriophora*, however, when the nematode invades an insect host, the bacteria switch to a lethal insect pathogen. Previous research has shown that *P. luminescens* contains a large number of genes encoding toxins and virulence factors, as well as molecules that assist the bacteria in evading the insect host humoral and cellular immune response [18, 19].

In the present study, we have shown that *chico* mutants have increased resistance to bacterial infection, they differentially regulate AMP gene transcripts, they have increased phenoloxidase activity but lower phagocytic ability, and they show no changes in their metabolic function. Our findings strongly suggest that *chico* participates in the immune response of *D. melanogaster* against pathogenic and non-pathogenic bacteria.

## Results

### Survival of *chico* mutants is unaffected upon bacterial infection

**Fig. 1:**
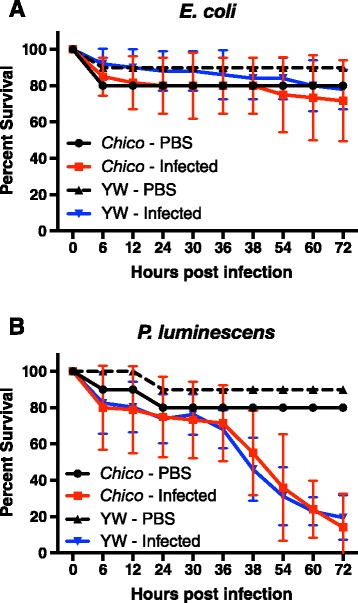
C*hico* mutants succumb to *P. luminescens* infection. Survival of 7-10 day old *Drosophila melanogaster chico* mutants and yellow white (yw) background control flies following intrathoracic injection with (**a**) non-pathogenic *Escherichia coli* bacteria (strain K12) or (**b**) pathogenic *Photorhabdus luminescens* bacteria (strain TT01). Injections with sterile PBS were used as septic injury controls. Survival was monitored for 72 h at 6-h intervals. Data analysis was performed using Log-Rank test (GraphPad Prism5 software) and the values are the percent survival of the infected flies. The means from three independent experiments are shown and error bars represent standard errors

We first investigated the survival response of *chico* mutants and their yellow white (yw) background control flies to infection by harmless *E. coli* bacteria. We found no significant differences in survival between the *chico* flies and their background controls following injection of *E. coli* (log-rank test, *P* > 0.05; Fig. 1a). We also found that intrathoracical injection of *P. luminescens* pathogenic bacteria resulted in substantial mortality of the flies; however, again there were no significant differences in the survival ability between the infected *chico* mutants and yw control flies (log-rank test, *P* > 0.05; Fig. 1b). These results suggest that loss of chico in *D. melanogaster* does not alter the survival phenotype of the flies against infection by the specific pathogenic or non-pathogenic bacteria.

### C*hico* mutants have increased resistance to bacterial infection

**Fig. 2:**
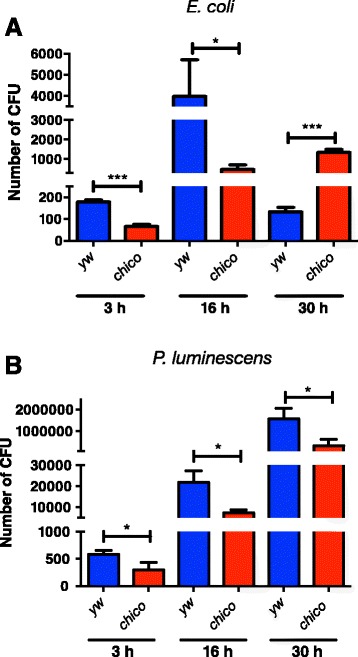
Persistence of *P. luminescens* bacteria decreases in *chico* mutants*. Escherichia coli* (strain K12) or *Photorhabdus luminescens* (strain TT01) bacteria were injected into 7-10 day old *Drosophila melanogaster chico* mutants and yellow white (yw) background control adult flies. Colony Forming Units (CFU) for (**a**) non-pathogenic *E. coli* and (**b**) pathogenic *P. luminescens* bacteria at 3, 16 and 30 h following bacterial infection were determined by quantitative PCR. Data analysis was performed by unpaired two-tailed *t*-test and significant differences are indicated by asterisks (***P < 0.0005; *P < 0.05). Bars show the means from three independent experiments and error bars represent standard deviation

To investigate whether *chico* mutants have altered resistance or tolerance following bacterial infection [20], we injected *E. coli* or *P. luminescens* cells into adult flies and estimated bacterial load over time. We found significantly higher numbers of *E. coli* cells in yw flies compared to *chico* mutants at an early (3 h) and relatively middle (16 h) time-point post infection (*P* < 0.0005 and *P* < 0.05, respectively; Fig. 2a); however, numbers of *E. coli* cells in *chico* flies were significantly higher than in yw individuals at a later (30 h) time-point (*P* < 0.0005; Fig. 2a). These results suggest that *chico* flies have increased resistance to *E. coli* at 3 and 16 h post infection, but decreased resistance to infection by these bacteria at 30 h post infection. For infections with the pathogen *P. luminescens*, we consistently found that yw flies contained significantly higher pathogen titers than *chico* mutant flies for each time-point tested in our experiments (*P* < 0.05; Fig. 2b). These results suggest that *chico* mutants have increased resistance to infection with the pathogen *P. luminescens*. Overall, these results indicate that *chico* can control resistance to pathogenic and non-pathogenic bacterial infections in *D. melanogaster*.

### C*hico* mutants have decreased transcript levels of AMP genes

**Fig. 3:**
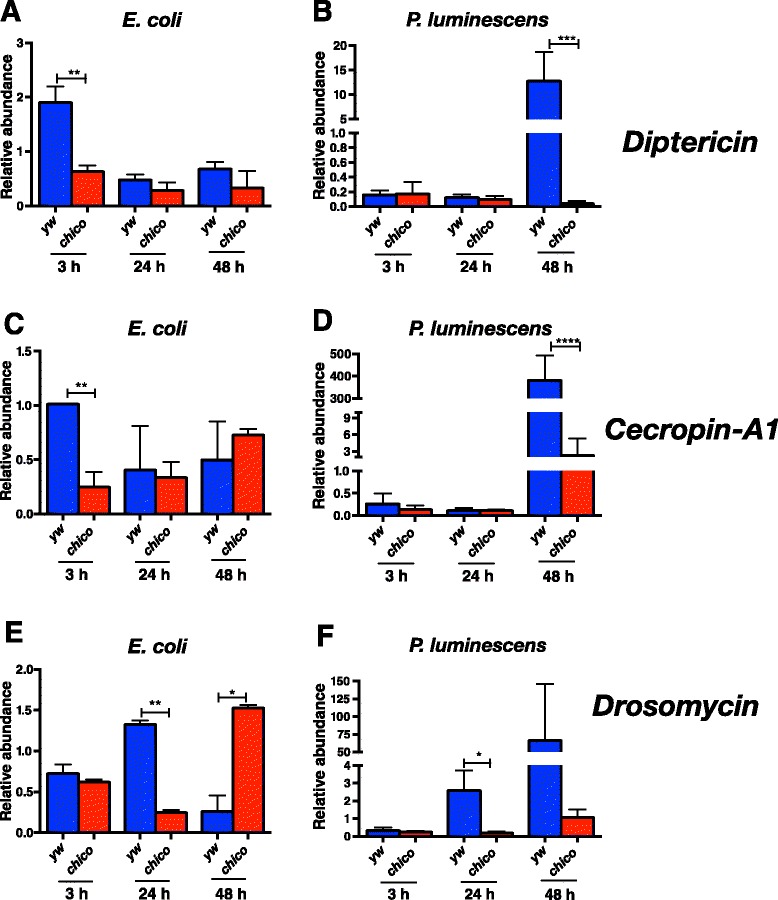
Transcript levels of antimicrobial peptide encoding genes are differentially regulated in *chico* mutants following bacterial infection. Gene transcript levels for (**a**, **b**) *Diptericin*, (**c**, **d**) *Cecropin-A1*, and (**e**, **f**) *Drosomycin* in 7-10 day old *Drosophila melanogaster chico* mutants and yellow white (yw) background control flies at 3, 24 and 48 h after infection with non-pathogenic *Escherichia coli* (strain K12) or pathogenic *Photorhabdus luminescens* (strain TT01) bacteria. Gene transcript levels are shown as relative abundance of transcripts normalized to gene *RpL32* and expressed as a ratio compared to flies injected with sterile PBS (negative control). Values represent the means from three biological replicates and error bars represent standard deviations. ****P < 0.0001; ***P < 0.001; **P < 0.01; *P < 0.05 (one way analysis of variance with a Tukey *post hoc* test, GraphPad Prism5 software)

To examine whether activation of immune deficiency (Imd) and Toll signaling is altered in *chico* mutants following infection with pathogenic or non-pathogenic bacteria [21], we estimated the transcript levels of AMP encoding genes in *D. melanogaster* flies injected by either *P. luminescens* or *E. coli* (Fig. 3). We found that *Diptericin* transcripts were significantly higher in yw than in *chico* flies at 3 h post infection with *E. coli* (*P* < 0.01; Fig. 3a), and there were no significant changes thereafter (*P* > 0.05; Fig. 3a). Infection with *P. luminescens* significantly upregulated *Diptericin* transcript levels in yw flies compared to *chico* mutants at 48 h post infection (*P* < 0.001; Fig. 3b), and there were no other significant changes at 3 and 24 h post infection with the pathogen (*P* > 0.05; Fig. 3b). Similarly, there were significantly higher mRNA levels of *Cecropin-A1* in yw controls than in *chico* mutants at 3 h post infection with *E. coli* (*P* < 0.01; Fig. 3c) and at 48 h post infection with *P. luminescens* bacteria (*P* < 0.0001; Fig. 3d), and no other significant changes in *Cecropin-A1* transcripts were observed for the rest of the time-points (*P* > 0.05; Fig. 3c and d). *Drosomycin* transcripts were significantly increased in yw compared to *chico* flies at 24 h after infection with *E. coli* or *P. luminescens* (*P* < 0.01; Fig. 3e and *P* < 0.05; Fig. 3f); however, *Drosomycin* transcripts were significantly higher in *Chico* mutants than in control individuals (*P* < 0.05; Fig. 3e). No significant changes in *Drosomycin* transcript levels between mutants and controls were found at any time-point after infection with these bacteria (*P* > 0.05; Fig. 3e and f). These results imply that Chico can regulate the transcriptional activation of the nuclear factor kappaB (NF-κB) immune signaling pathways in *D. melanogaster* in response to infection by certain pathogenic and non-pathogenic bacteria.

### C*hico* mutants have increased melanization and phenoloxidase activity

**Fig. 4:**
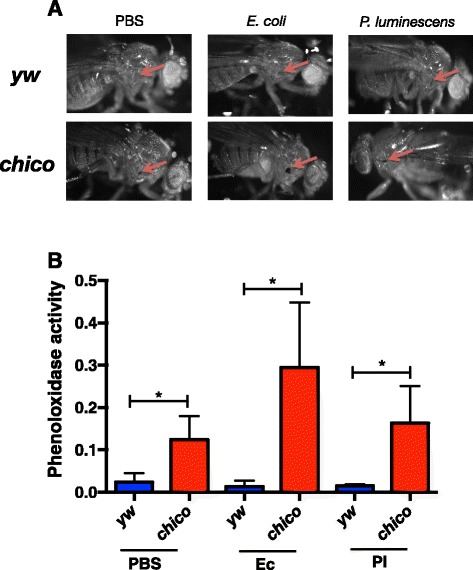
Melanization and phenoloxidase activity are elevated in *chico* mutants. **a** Melanization of the wound site is shown at 40x magnification 3 h after injection with non-pathogenic *Escherichia coli* (strain K12), pathogenic *Photorhabdus luminescens* (strain TT01) bacteria, or sterile PBS in 7-10 day old *Drosophila melanogaster chico* mutants and yellow white (yw) background control flies. **b** Phenoloxidase activity in the hemolymph plasma of *chico* mutant and yw control flies injected with PBS, *E. coli* (Ec), or *P. luminescens* (Pl) as measured by the optical density at 492 nm after incubation with L-Dopa. Values are shown as the mean of three independent experiments with error bars representing standard deviations. *P < 0.05 (unpaired two-tailed *t*-test, GraphPad Prism5 software)

The in vivo melanization response of yw control and *chico* mutant flies was examined superficially by visually inspecting the wound site 3 h after injection of bacteria or phosphate buffered saline (PBS, septic injury control). The darkness of the wound is associated with relative melanization activity and therefore phenoloxidase activity, with a darker spot indicating a higher degree of melanization. We found that melanization of the wound site following injection with bacteria or PBS was more intense in the *chico* flies compared to background controls (Fig. 4a). The degree of phenoloxidase activity in response to infection or wounding was assessed in *chico* mutants and yw flies by collecting hemolymph 3 h after infection and measuring the capacity of this extract for the oxidation of L-Dopa, which results in a color change that is quantifiable by optical density (OD) [22]. Injection with *E. coli, P. luminescens* bacteria*,* or PBS resulted in significantly higher phenoloxidase activity in *chico* mutants as compared to yw background flies for all three treatments (*P* < 0.05; Fig. 4b). Together, these results demonstrate a consistently higher level of phenoloxidase activity in *chico* mutant flies as compared to their yw background controls, which suggests that *chico* can act as regulator of the phenoloxidase antibacterial immune response in *D. melanogaster*.

### C*hico* mutants have decreased phagocytosis ability

**Fig. 5:**
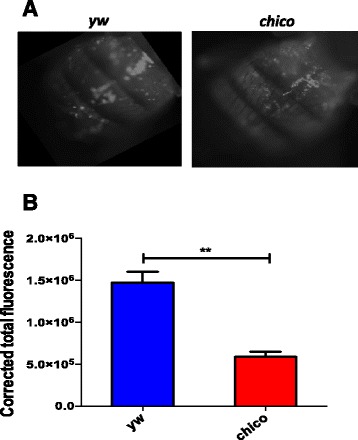
Phagocytosis decreases in *chico* mutant flies. **a** Representative images of phagocytosis in *Drosophila melanogaster chico* mutants and yellow white (yw) background control 7-10 day old adult flies at 60 min after injection of pHrodo-labeled *Escherichia coli* particles. Images were taken using fluorescence microscopy at 10x magnification. **b** Corrected total fluorescence in *chico* mutant and control flies (*n* = 7) at 60 min following injection of pHrodo-labeled *E. coli* particles. Images were processed in ImageJ and corrected total fluorescence was estimated by measuring relative amounts of fluorescence, which involved estimations of the resulting area, mean fluorescence of background and integrated density. Phagocytosis experiments were repeated three times. Values shown are means and error bars represent standard deviations **P < 0.01 (unpaired two-tailed *t*-test, GraphPad Prism5 software)

To evaluate whether absence of *chico* leads to changes in the phagocytic ability of *D. melanogaster* flies, *chico* mutants as well as background control flies were injected with inactivated unopsonized fluorogenic pHrodo *E. coli* particles. These particles are labeled with a pH sensitive dye that fluoresces in acidic environment. Thus, when pHrodo-labeled bacteria are phagocytosed by the hemocytes and exposed to lysosomal acidic environment, the cells emit red fluorescence [23, 24]. Hence, phagocytosis around the periostial regions of the heart can be imaged through the dorsal surface of live flies. At 1 h post bacterial injection, we observed fewer fluorescent *E. coli* bioparticles in *chico* than in yw background control flies (Fig. 5a). Quantification of fluorescence confirmed that phagocytosis of *E. coli* particles in *chico* flies was significantly lower compared to control individuals (*P* < 0.01, Fig. 5b). These results suggest that inactivation of *chico* drastically affects phagocytosis of bacteria in *D. melanogaster*.

### C*hico* mutants do not show changes in metabolic functions

**Fig. 6:**
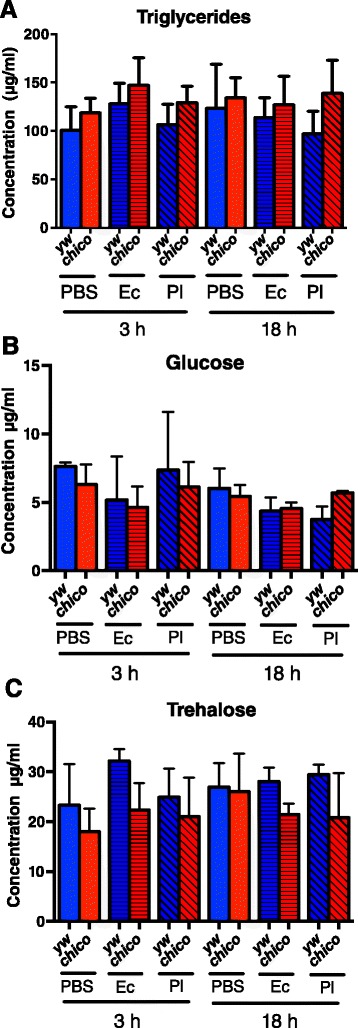
Mutant flies for *chico* show no changes in metabolism upon infection with bacteria. **a** Triglyceride, (**b**) Glucose and (**c**) Trehalose concentrations in *Drosophila melanogaster chico* mutant and yellow white (yw) background control 7-10 day old adult flies at 3 and 18 h post-injection with PBS buffer, *Escherichia coli* (Ec, strain K12) or *Photorhabdus luminescens* (Pl, strain TT01) bacteria. Triglyceride, glucose and trehalose contents were estimated based on their respective standard curves. The experiment was repeated three times. Values shown are means and error bars represent standard deviations (one way analysis of variance with a Tukey *post hoc* test, GraphPad Prism5 software)

To test whether the absence of *chico* affects the metabolic function in *D. melanogaster* adult flies in response to bacterial infection, *chico* mutants and their background control flies were injected with *E. coli*, *P. luminescens* or PBS. Total protein, triglyceride, glucose and trehalose concentrations were estimated at 3 and 18 h post-injection. We observed no significant differences in triglyceride (Fig. 6a), glucose (Fig. 6b) and trehalose (Fig. 6c) concentrations between uninfected *chico* flies and their background yw controls. Similarly, no major differences in triglyceride, glucose and trehalose amounts were found in flies previously infected with *E. coli* or *P. luminescens* bacteria (Fig. 6a-c). These results evidently demostrate that *chico* is not invloved in the control of metabolic processes in *D. melanogaster* in the absence or presence of infection with the specific bacteria.

## Discussion

Here we have tested the immune response of *D. melanogaster* flies mutant for the insulin receptor substrate Chico against pathogenic and non-pathogenic bacteria. We have chosen to use a professional insect pathogen, the virulent bacterium *P. luminescens*, the genome of which contains a large number of genes coding for proteins with high insecticidal activity [25, 26]. As a direct comparison to the Gram-negative *P. luminescens*, we used a non-pathogenic strain of *E. coli* that is similar to *P. luminescens* at the genome level, but is not virulent to wild-type flies [27]. We have found that although *chico* mutants survive equally a challenge by *P. luminescens* or *E. coli* bacteria compared to controls, they contain less bacteria during the infection, they express AMP-encoding genes at low levels, they activate phenoloxidase and melanization responses at high levels, and they are less able to phagocytose bacterial bioparticles.

Current survival results demonstrate that suppression of insulin signaling in *D. melanogaster* by mutation of the insulin receptor substrate Chico does not affect the survival of the mutant flies upon bacterial infection. These findings are not in line with results from a previous study reporting nearly three-fold increased survival for *Chico* homozygous and heterozygous flies following infection with two pathogenic bacteria, the Gram-negative *Pseudomonas aeruginosa* and the Gram-positive *Enterococcus faecalis* [16]. One possibility that could explain the discrepancy between the survival results in the two studies could be the use of flies heterozygous and homozygous for the *Chico* mutation in the previous study, whereas in our study the *chico* flies have the *chico* hypomorphic but not null alleles [28]. A second possibility to take into account is that the previous study involved pathogenic bacteria that are not natural pathogens of insects [29], whereas the current study involves infection with the insect pathogenic bacterium *P. luminescens*. Therefore the responses of the *chico* mutant flies to pathogenic challenges may vary substantially. In addition, the previous study omitted an estimate of the numbers of bacterial cells in the *chico* mutants during the course of infection. Estimating the bacterial load in an infected host is important because we can distinguish between resistance (the ability of the host to control pathogen load) and tolerance (the ability of the host to withstand the damage and consequences at a given pathogen load) [30]. Here we have investigated for the first time bacterial load in the long-lived *chico* mutant flies. We have found that although both fly strains succumb similarly to infection by *P. luminescens* and they are both unaffected by infection with *E. coli*, the *chico* mutants contain fewer pathogenic or non-pathogenic bacteria than their background controls. Therefore we have concluded that inactivation of *chico* can restrain the growth of certain bacteria and confer immune resistance to *D. melanogaster*.

To understand the reduced growth of *P. luminescens* and *E. coli* in *chico* mutant flies, we first estimated the activation of the NF-κB signaling pathways Imd and Toll that regulate the expression of AMP genes in *D. melanogaster* [31, 32]. The expression of certain AMP genes in *D. melanogaster* is used as an indicator of the activation of the humoral immune response against microbial infections [33]. Therefore we analyzed the transcript levels of *Diptericin* and *Drosomycin* genes that are convenient read-outs for Imd and Toll pathway activation [34]. Although our experiments involved infections with two Gram-negative bacteria, we assessed *Drosomycin* gene transcript levels because this AMP can be reasonably stimulated from the Imd pathway in the systemic immune response of the fly [35]. We further tested *Cecropin-A1* gene transcripts because this AMP can act against different types of bacteria [36]. Here we were expecting to find increased transcriptional activation of AMP genes in *chico* infected mutants that would elucidate the decreased replication of bacteria in these flies. However, we have unexpectedly found no changes in AMP gene transcripts between *chico* mutants and controls infected with *E. coli* or *P. luminescens* or in some cases there were reduced mRNA levels of AMP genes in *chico* flies infected with either bacterial species. Interestingly, there was only one case in which *Drosomycin* transcript levels were higher in *chico* flies than in background controls following infection with the pathogen *P. luminescens*. We can conclude from these findings that inactivation of *chico* in *D. melanogaster* does not affect, or in some cases reduces, AMP transcript levels in response to *E. coli* or *P. luminescens* challenge, an outcome that does not affect the survival of the mutant flies, but it can limit the growth of these bacteria.

We then determined the melanization response and phenoloxidase activity, which overlap the humoral and cellular arms of the immune response in *D. melanogaster* and form rapid immune reactions upon invasion of foreign microbes into the hemolymph of the fly [37, 38]. A few hours after injection of the bacteria, we measured both qualitative differences in melanization at the site of injection as well as quantitative changes in phenoloxidase enzyme activity in the hemolymph of the infected flies. In all three treatments there were visibly larger size melanin spots at the site of injection on the cuticle of the *chico* flies compared to the background controls, and increased melanization was accompanied by elevated levels of phenoloxidase activity in the mutants. The fact that phenoloxidase activity levels are higher in *chico* mutants than in yw controls injected with PBS probably suggests that *chico* flies possess high amounts of endogenous enzyme in its active form, which can further increase upon bacterial infection. The molecular/biochemical basis to interpret the increased levels of phenoloxidase in *chico* mutants is currently unknown and it will form a topic for future investigation. We also noticed that phenoloxidase activity remains at low levels in control flies challenged with *P. luminescens*. This could be due to the ability of this pathogen to suppress phenoloxidase activity in *D. melanogaster*, as it was shown previously in other insects [39–41]. Interestingly, this is not the case in *P. luminescens* infected *chico* mutants where phenoloxidase activity remains at high levels. This could imply that the pathogen is unable to interfere with the activation of the prophenoloxidase cascade in the absence of *Chico*. We were not surprised to find low phenoloxidase activity in yw flies infected by *E. coli*; we have shown recently that this strain exhibits low phenoloxidase response compared to other reference laboratory fly strains [42].

We further estimated the *D. melanogaster* cellular response, which is mainly governed by the function of phagocytosis that involves the activity of circulating macrophage-like insect blood cells called plasmatocytes [43]. We avoided using stained *P. luminescens* bacteria in these experiments because this pathogen is able to suppress the insect cellular immune response by producing factors that inhibit phagocytosis; this forms a strategy for promoting pathogen persistence and replication in the host [44]. Although our expectation was that reduced bacterial persistence in the *chico* mutants would be probably due to increased cellular immune activity in these flies, increased phagocytosis does not seem to account for the lower levels of viable cells since fewer phagocytic events were observed in the mutants.. The sharp decrease in the ability of *chico* mutants to engulf inactive *E. coli* particles implies that for a reason that is currently unkown, phagocytosis is disrupted in *chico* deficient *D. melanogaster* flies. This could be either the result of substantial reduction in the number of plasmatocytes present in the hemolymph of *chico* mutants or a decline in bacterial uptake by plasmatocyte cells, or a combination of these two possibilities. Alternatively, if the bacteria are eliminated by the phenoloxidase/melanization response, then there would be fewer cells available to be phagocytosed. Thus, the fewer phagocytic events in *chico* flies could reflect fewer available bacteria and may not mean that the mutants have a reduced phagocytic ability.

We have found increased resistance of *chico* mutants to *E. coli* and *P. luminescens* because these flies diminish the burden of bacteria during infection. Given the similarity in survival between the *chico* mutants and their background controls, the observed elevation in resistance could be possibly balanced by a reduction in tolerance that could in turn cause disease symptoms in *chico* flies. Therefore we examined the metabolic activity of *chico* infected and uninfected mutants by measuring the amount of glucose and trehalose produced in these flies. In addition, we tested triglyceride concentrations because lipid is the main component of insect fat body cells, and more than 90 % of stored lipid is triglyceride [45, 46]. Our results clearly demonstrate that *chico* flies do not exhibit metabolic changes upon infection with pathogenic *P. luminescens* or non-pathogenic *E. coli*, and the increased resistance of the mutants to infection by these bacteria is not accompanied by changes in energy stores.

## Conclusions

In this study we have shown that the *D. melanogaster* long-lived *chico* mutant flies have increased resistance to infection by two bacteria; the pathogen *P. luminescens* and a non-pathogenic strain of *E. coli*. To understand the mechanism behind the increased resistance in *chico* mutant flies, we examined the three resistance mechanisms of the *D. melanogaster* innate immune response that are important for limiting microbial growth: AMP production, phenoloxidase activity/melanization, and phagocytosis [47, 48]. Although AMP gene transcripts and phagocytosis rates were unaffected or lower in *chico* flies than in controls, *chico* mutants showed elevated levels of melanization and phenoloxidase activity, which could potentially contribute to higher resistance against the two bacteria. These results reveal that *chico* plays a distinct regulatory role in the *D. melanogaster* immune response against certain bacterial infections. Taken together, the current study indicates that research in model systems, such as *D. melanogaster*, can provide critical evidence for the interaction between immunity and ageing mechanisms, and whether altering one process can affect the other. Furthermore, studies using long-lived *D. melanogaster* mutants in immunity research will significantly serve to identify key players involved in the regulation of the immune response in vertebrate animals, perhaps even in humans.

## Methods

### Fly and bacterial strains

*Chico*^*KG00032*^ mutant strain and its background yw strain were used in all experiments. Flies were purchased from Blomington Stock Center and grown at 25 °C on standard diet, as previously described [49]. Mutant flies for *chico* were backcrossed into the yw background controls for over six generations. Equal number of young male and female 7-10 days old adult flies were used for infections with bacteria. All fly injections were perfromed during the morning hours.

The insect pathogenic bacterium *Photorhabdus luminescens* subsp. laumondii (strain TT01) and the non-pathogenic K12 strain of *Escherichia coli* were used for fly infections. Bacterial cultures were grown for 18 h at 30 °C on a rotary shaker at 210 rpm and then prepared for infections as described before [49].

### Fly survival

Adult flies of the *chico* mutant and its background control were anesthetized briefly with carbon dioxide and then injected into the thorax with 18.4 nl containing approximately 100-300 colony-forming units (CFU) of *P. uminescens* or *E. coli* using a microinjector (Nanoject II, Drummond Scientific) and glass capillaries. Injection with 1x sterile PBS served as septic injury control. All flies were placed into newly prepared vials post-injection and kept in an incubator at 25 °C. Infected and uninfected flies were observed every 6 h for 72 h post injection and the number of dead individuals was recorded. Two replicates of 10 flies were used for each experimental condition and each experiment was repeated three times.

### Bacterial load

Four adult flies from each strain were injected with *E. coli* or *P. luminescens* and the flies were subsequently frozen at 3, 16 and 30 h post infection. DNA was extracted using the DNeasy Blood and Tissue kit (Qiagen) following the manufacturer’s instructions. DNA concentrations were measured using a NanoDrop. Each PCR reaction included 10 μl of EXPRESS SYBR® GreenER with Premixed ROX (Invitrogen), 10 μM of each forward and reverse primer sets and 300 ng of each DNA sample. The primers used were Mcf-1 (*P. luminescens*), Forward: TTGGCGGGGTGGTAGTCG and Reverse: CAGTTCAGCTTCCTTCTCTAA; and 16S rRNA (*E. coli*), Forward: GGAAGAAGCTTGCTTCTTTGCTGAC and Reverse: AGCCCGGGGATTTCACATCTGACTTA. Cycling conditions were 50 °C for 2 min, 95 °C for 2 min, 40 cycles of 95 °C for 15 s and an annealing step of 61 °C for 15 s. All samples were run in technical duplicates and three independent experiments were carried out. Bacterial load (numbers of CFU) was estimated from the standard curves that were generated for *E. coli* and *P. luminescens*.

### Gene transcript levels

Infected and PBS-injected *chico* mutant and yw background control flies were collected at 3, 24 and 48 h post infection and stored in a -80 °C freezer. Four flies were used for each experimental condition. Total RNA was extracted using the PrepEase RNA spin kit (Affymetrix USB) following the manufacturer’s instructions. cDNA synthesis was carried out using a MultiScribe Reverse Transcriptase Kit (Applied Biosystems), random primers and 0.1 μg of RNA template as starting material in a total reaction volume of 20 μl following the manufacturer’s protocol. Resulting cDNA samples were diluted 1:10 in nuclease-free water and 1 μl was used as template for quantitative RT-PCR experiments. These were performed using the EXPRESS SYBR® GreenER kit with Premixed ROX (Invitrogen) in twin.tec real-time PCR 96-well plates on a Mastercycler ep realplex^2^ (Eppendorf). Primers were purchased from Eurofin MWG Operon. Primer sequences for *Diptericin* (CG12763) *Drosomycin* (CG10146), and *Cecropin-A1* (CG1365) have been given before [48]. The reactions were carried out in a total volume of 20 μl under the following conditions: 50 °C for 2 min, 95 °C for 2 min, 40 cycles of 95 °C for 15 s and an annealing step for 45 s. mRNA values were normalized to mRNA values of the control housekeeping gene *RpL32* (CG7939) [50]. Normalized data were used to quantify the relative level of a given mRNA as previously described [49]. Data are presented as the ratio between infected versus PBS injected flies (negative controls). Technical duplicates were run for each sample and set of primers and each experiment was replicated three times.

### Phenoloxidase activity and melanization

Twenty flies were injected with bacteria or PBS as mentioned above and hemolymph samples were extracted 3 h after injection. First, the flies were placed on a 10 μM spin column (ThermoFisher Scientific) containing 30 μl of 2.5X Protease inhibitor (Sigma) and then they were covered with five 4 mm glass beads (VWR). Spin columns were centrifuged at 13,000 rpm for 20 min at 4 °C. Protein concentrations were adjusted using a Pierce™ BCA Protein assay kit (ThermoFisher Scientific) following the manufacturer’s instructions. A total volume of 40 μl containing a mixture of 15 μg of protein (diluted in 2.5x protease inhibitor) with 5 mM Cacl_2_ was added to 160 μl of L-DOPA solution (15 mM in phosphate buffer, pH 6.6). After 30 min of incubation in the dark at 29 °C, the OD at 492 nm was measured for each sample against a blank control. Each assay was carried out in biological duplicates and each experiment was repeated three times. Melanization spots on the thorax of the challenged flies were visualized at 3 h post injection using Nikon SMZ18 microscope with Zyla (ANDOR) 5.5 camera. Images were analyzed using Nikon Software Suite.

### Phagocytosis estimation

Seven flies from each strain were injected with 50 nL of 1 mg/ml pHrodo labeled *E. coli* (Molecular Probes) and allowed to phagocytose at room temperature for 60 min. The flies were fixed ventrally on a glass slide using clear nail paint. Fluorescent images of the dorsal surface were obtained using Nikon ECLIPSE N*i* microscope (10x magnification) fitted with Zyla (ANDOR) 5.5 camera. The images were analyzed with ImageJ software. The relative amounts of fluorescence were measured by estimating the resulting area, mean fluorescence of background and integrated density. Corrected total fluorescence was determined using the following equation: Corrected total fluorescence (CTF) = Integrated Density (ID)– (Area * Mean fluorescence of background). The experiment was repeated three times on three different days.

### Metabolic activity assays

Five adult flies from each strain were injected with *E. coli*, *P. luminsecens*, or 1x sterile PBS and samples were collected at 3 and 18 h post injection. Samples were processed using a previously published protocol [51]. Protein quantification was performed using the Pierce™ BCA Protein assay kit (ThermoFisher Scientific) following the manufacturer’s instructions. The microtiter plate was covered and placed on a shaker for 30 s followed by incubation at 37 °C for 30 min. Absorbance was measured at 562 nm on a Synergy HT plate reader (BioTek). Protein concentrations of samples were calculated from the standard curve. For estimating metabolic functions in infected and PBS-injected flies, the protein concentrations of the samples were normalized. Standard curve for triglyceride estimation was made using the Glycerol Standard Solution (Sigma). Triglyceride content was measured at 37 °C and 520 nm using the Infinity™ Triglycerides Liquid Stable Reagent (ThermoFisher Scientific). Glucose standard curve was constructed using the Glucose Standard Solution (Sigma) and the trehalose standard curve was made using Trehalose Dihydrate (Sigma). Free glucose in the samples was measured at 340 nm using the HK reagent (Sigma). Trehalose measurement was obtained by subtracting the absorbance of free glucose from samples that were digested with trehalase. Trehalose content was then calculated using the trehalose standard curve. All samples and standards were run in duplicates and three independent experiments were carried out.

### Statistical analysis

Statistical analysis was performed using the GraphPad Prism5 software. Analysis of survival data was performed using Log-Rank test (Mantel-Cox). *P* values below 0.05 were considered statistically significant. For gene transcript levels and metabolic activity estimation, data were analyzed using a one-way analysis of variance (ANOVA) with a Tukey post hoc test for multiple comparisons. For bacterial load, phenoloxidase activity and phagocytosis estimation, samples were analyzed using two-tailed *t*-test.

## Abbreviations

AMP: antimicrobial peptide
ANOVA: analysis of variance
CFU: colony forming units
CTF: corrected total fluorescence
ID: integrated density
IIS: insulin/insulin-like growth factor signaling pathway
Imd: immune deficiency
NF-κB: nuclear factor kappaB
OD: optical density
PBS: phosphate buffered saline
yw: yellow white
